# Determining the optimal diagnostic and risk stratification approaches for people with hypertension in two rural populations in Kenya and The Gambia: a study protocol for IHCoR-Africa Work Package 2

**DOI:** 10.3310/nihropenres.13509.2

**Published:** 2024-05-17

**Authors:** Alexander D Perkins, Juliet Otieno Awori, Modou Jobe, Ruth K Lucinde, Meike Siemonsma, Robinson Oyando, David A Leon, Emily Herrett, Andrew M. Prentice, Anoop SV Shah, Pablo Perel, Anthony Etyang, The IHCoR-Africa Collaborators

**Affiliations:** 1 Department of Non-communicable Disease Epidemiology, London School of Hygiene & Tropical Medicine, London, WC1E 7HT, UK; 2 Department of Epidemiology and Demography, KEMRI Wellcome Trust Research Programme, Kilifi, Kenya; 3 Medical Research Council Unit The Gambia at LSHTM, Banjul, The Gambia; 4 Health Economics Research Unit, KEMRI-Wellcome Trust Research Programme, Nairobi, Kenya

**Keywords:** Hypertension, Kenya, The Gambia, Blood pressure, Hypertension mediated organ damage, Community

## Abstract

**Background:**

Sub-Saharan Africa (SSA) has one of the highest prevalences of hypertension worldwide. The impact of hypertension is of particular concern in rural SSA, where access to clinics and hospitals is limited. Improvements in the management of people with hypertension in rural SSA could be achieved by sharing diagnosis and care tasks between the clinic and the community. To develop such a community-centred programme we need optimal approaches to identify and risk stratify patients with elevated blood pressure. The aim of the study is to improve the evidence base for diagnosis and risk estimation for a community-centred hypertension programme in two rural settings in SSA.

**Methods:**

We will conduct a cross-sectional study of 1250 adult participants in Kilifi, Kenya and Kiang West, The Gambia. The study has five objectives which will determine the: (1) accuracy of three blood pressure (BP) measurement methods performed by community health workers in identifying people with hypertension in rural SSA, compared to the reference standard method; (2) relationship between systolic BP and cardiovascular risk factors; (3) prevalence of hypertension-mediated organ damage (HMOD); (4) accuracy of innovative point-of-care (POC) technologies to identify patients with HMOD; and (5) cost-effectiveness of different combinations of BP and HMOD measurements for directing hypertension treatment initiation.

**Expected findings:**

This study will determine the accuracy of three methods for community BP measurement and POC technologies for HMOD assessment. Using the optimal methods in this setting it will estimate the prevalence of hypertension and provide the best estimate to date of HMOD prevalence in SSA populations. The cost-effectiveness of decision-making approaches for initiating treatment of hypertension will be modelled. These results will inform the development of a community-centred programme to improve care for hypertensive patients living in rural SSA. Existing community engagement networks will be used to disseminated within the research setting.

## Plain English Summary

Many people live with high blood pressure in sub-Saharan Africa. In this region, the proportion of people with high blood pressure is one of the highest in the world. However, few people with high blood pressure are treated and this can lead to serious medical issues and even death. This is particularly true in rural areas where treatment and understanding of blood pressure is lower than in cities.

There are many reasons why high blood pressure is a major health problem in rural sub-Saharan Africa, such as a lack of clear symptoms; less access to healthcare; and limited time to travel to clinics for care. One option for improving the management of blood pressure is to use a community-centred approach, where care is brought into the community making it easier to access.

To bring care into the community, we need to find out what is the best way for community health workers to identify who needs to be treated. Standard techniques may not be useful in a rural community and could require too many resources to make them practical. This study aims to determine what is the best way to identify high blood pressure and related health complications in a community setting.

The study will take place across two sites: one in Kilifi, Kenya and the other in Kiang West, The Gambia. We will enrol 1250 participants, with 625 in each country. The people living in these areas have been involved in the design of this study through community engagement and have helped identify the need for improving how blood pressure is treated in a rural areas. Throughout this study, we will continue to meet with the communities. Once the study is completed, we will use our strong links with the communities, healthcare providers and policymakers to share the results.

## Introduction

Hypertension is the single risk factor that accounts for the highest number of deaths (10.8 million) globally ^1, 2^. Sub-Saharan Africa (SSA) has one of the highest estimated prevalences of hypertension worldwide, and in 2019 hypertension was implicated in almost 700,000 deaths in SSA – double the number in 1990 ^1, 2^. In SSA, hypertension occurs at younger ages, is more severe, remains very poorly controlled, and is more likely to cause complications including heart failure, stroke, kidney disease and premature death than in other regions ^3, 4^.

The impact of hypertension is of particular concern in rural SSA where 60% of the region’s 1.1 billion population live. While hypertension prevalence is as high in rural as in urban settings, hypertension awareness, treatment and control is lower in rural settings ^5– 7^.

The NIHR Global Research Group on Improving Hypertension Control in Rural Sub-Saharan Africa ( IHCoR-Africa) has been set-up to address these pressing issues and the study described here is part of that effort.

To significantly reduce hypertension-related burden, there is a need to improve the whole hypertension control cascade from awareness (screening and diagnosis) to management (risk stratification and treatment) and control (monitoring, adherence, and referral) ^8^. The barriers to effective hypertension management in rural SSA are multiple and complex. At an individual-level, key issues are the asymptomatic nature of hypertension, a lack of understanding of long-term risk, financial impact of taking time away from work to seek treatment, caring responsibilities, and misconceptions about the benefits of pharmocotherapy ^9– 13^. At the provider-level *,* barriers include poor communication between providers and patients, lack of skills and competencies, poor infrastructure, and lack of adequate referral systems for care ^14^. Finally, system level barriers include poor access to health care facilities (caused by overcrowding and long distances between homes and clinics); irregular access to medications; lack of affordable treatments including anti-hypertensive drugs; and underinvestment in health service capacity ^15^.

There is growing evidence from other regions where access to medical care is limited that community health workers (CHWs) can play a key role in managing hypertension and its consequences ^16– 18^. The HOPE-4 study, conducted in Colombia and Malaysia, showed that a community-centred intervention including screening, diagnosis and treatment with combination pharmacotherapy administered by CHWs and supported by electronic decision support tools improved blood pressure control ^19^. Similarly, the COBRA study, in Southeast Asia also showed improved hypertension management delivered using a community-centred intervention by CHWs ^20^. This evidence base has led organizations such as the Pan African Society of Cardiology (PASCAR) to advocate for community-centred approaches to improve hypertension management in SSA ^21^.

However, equivalent studies in rural SSA are lacking, resulting in limited evidence for clinical recommendations in these settings. Specifically, it is unclear whether recommended approaches to the diagnosis of hypertension and estimation of cardiovascular disease (CVD) risk are appropriate and effective for use in these settings. Clinical guidelines, developed from high-income country studies, ubiquitously incorporate these two aspects when guiding decision making on treatment, including pharmacotherapy ^22, 23^. International guidelines recommend repeated clinic-based blood pressure (BP) measurement such as Automated BP Measurement (ABPM) *,* which can be performed with (attended; aABPM) or without (unattended; uABPM) a health worker present, to mitigate for white coat hypertension. Home Blood Pressure Measurement (HBPM) or 24-hour ambulatory blood pressure monitoring (24-hr ABPM) are also deemed to have a role ( Table 1). However, these international recommendations might not be accurate, feasible or cost-effective in SSA and require further investigation ^24, 25^.

**Table 1.** Overview of the methods used to measure blood pressure.

Method	Abbreviation	Brief description
Reference standard		
24-hour Ambulatory BP Measurement	24-hr-ABPM	24-hr-ABPM measures blood pressure on a continuous basis for 24-hours at fixed time intervals. The device will be attached to the body and will be carried by the participant for 24-hours after which the CHWs will transfer data to a study computer.
Alternative methods		
Attended Automated Blood Pressure Measurement	aABPM	CHWs will measure BP using an automated machine in the home of the participant. Measurement procedures will follow international recommendations. CHWs and participants will be blinded to readings until both the aABPM and uABPM measurements are complete.
Unattended Automated Blood Pressure Measurement	uABPM	Using the same BP machine as the aABPM, the participant’s BP will be measured without the CHW present in the room. Once aABPM and uABPM measurements are completed, the CHW will enter the results into study database.
Home BP Measurement	HBPM	The participant receives a digital blood pressure machine at home and is asked to record their blood pressure every morning and evening for 7 consecutive days. CHWs will provide instructions and the device will automatically store readings which CHWs will download at the end of the 7 day period. Literate participants will be asked to write down measurements in supplied diaries.

International guidelines use an absolute risk approach to inform treatment. For example, although all guidelines recommend starting anti-hypertensives for a person with a BP over 160/100 mmHg, the International Society of Hypertension guideline only recommends anti-hypertensives for someone with a BP over 140/90 mmHg, when they are at high CVD risk as determined by risk scoring ^26^. In rural SSA, the suitability of existing risk scores is unclear. First, risk scores were developed from populations where the prevalence of known risks such as smoking and high cholesterol is much higher than in rural SSA ^27– 29^. Second, compared to high-income countries, most studies in SSA show a substantial burden of hypertension on younger age groups but the age-component of current risk scores classify younger individuals as at minimal risk. This is particularly pertinent given that age-adjusted cardiovascular event rates are higher in low- and middle-income countries than in high-income settings ^30– 32^. Third, current risk scores do not consider region-specific factors in SSA, including co-existing chronic communicable disease (HIV and malaria), or exposure to environmental factors (indoor air pollution) ^33– 35^. Finally, genetic factors could be contributing to the different epidemiological picture of hypertension and CVD risk in SSA ^36, 37^. Therefore, novel approaches to risk estimation are required.

One strong candidate that may have a key role in optimising risk stratification in rural SSA is hypertension-mediated organ damage (HMOD), which includes subclinical damage to organs including the heart, eyes, and kidney among other organs. HMOD has been reported to occur at younger ages and be more prevalent in SSA population ^38, 39^. Assessment of HMOD is recommended by clinical guidelines and is strongly associated with CVD among people with hypertension ^22, 23, 26^. However, significant challenges persist to measuring HMOD in routine practice, including the clinical resources required for echocardiograms, electrocardiograms, retinal imaging and laboratory blood analysis. Novel point-of-care (POC) diagnostic tools may remove this barrier but have not been validated in this setting.

A key unaddressed challenge is how to embed any new techniques for BP and HMOD measurement within the local health system, especially when CHWs are given more responsibilities without a parallel increase in resources and clinical supervision. To be feasible and sustainable, a community-centred approach to managing hypertension must take account of the health system context.

This paper reports the design of one study that is part of the wider NIHR IHCoR-Africa Group. IHCoR-Africa will also conduct another study addressing the health system challenges and patient experiences will be reported separately. The findings from both studies will contribute to the development of a new community-based intervention for improving hypertension management in rural SSA.

This study has been developed through an equitable collaborative partnership between the London School of Hygiene & Tropical Medicine (LSHTM; UK); the Kemri-Wellcome Trust Research Programme (KWTRP; Kenya) and the MRC Unit the Gambia (MRCG; the Gambia). This partnership was formed during the proposal phase and involved preparatory public and participant involvement and engagement to define the research objectives. Protocol drafting was supported by weekly meetings with representatives from all three partners and included two in person development meetings: November 2022 in Kilifi, Kenya and February 2023 in Fajara, the Gambia. The structure of the partnership enabled all members to have meaningful input in the design of the study.

## Methodology

### Patient and Public Involvement

This study has a strong emphasis on the involvement of the patients and the public. Before protocol development, the study was discussed with community leaders in the study areas so they could suggest changes and discuss how to involve the community. The IHCoR-Africa project has been presented to local departments of health in Kilifi and Gambia, and their recommendations taken into consideration. Throughout the course of the study we will meet with CHWs and patient groups, to understand their experiences as the project is implemented. The collection of data will be mainly performed by CHWs, who are members of the studied communities. After the conclusion of the study, we will involve CHWs and other members of the community in developing an intervention that should improve the detection, treatment, and control of hypertension in rural Gambia and Kenya.

### Study setting

**Figure 1.**
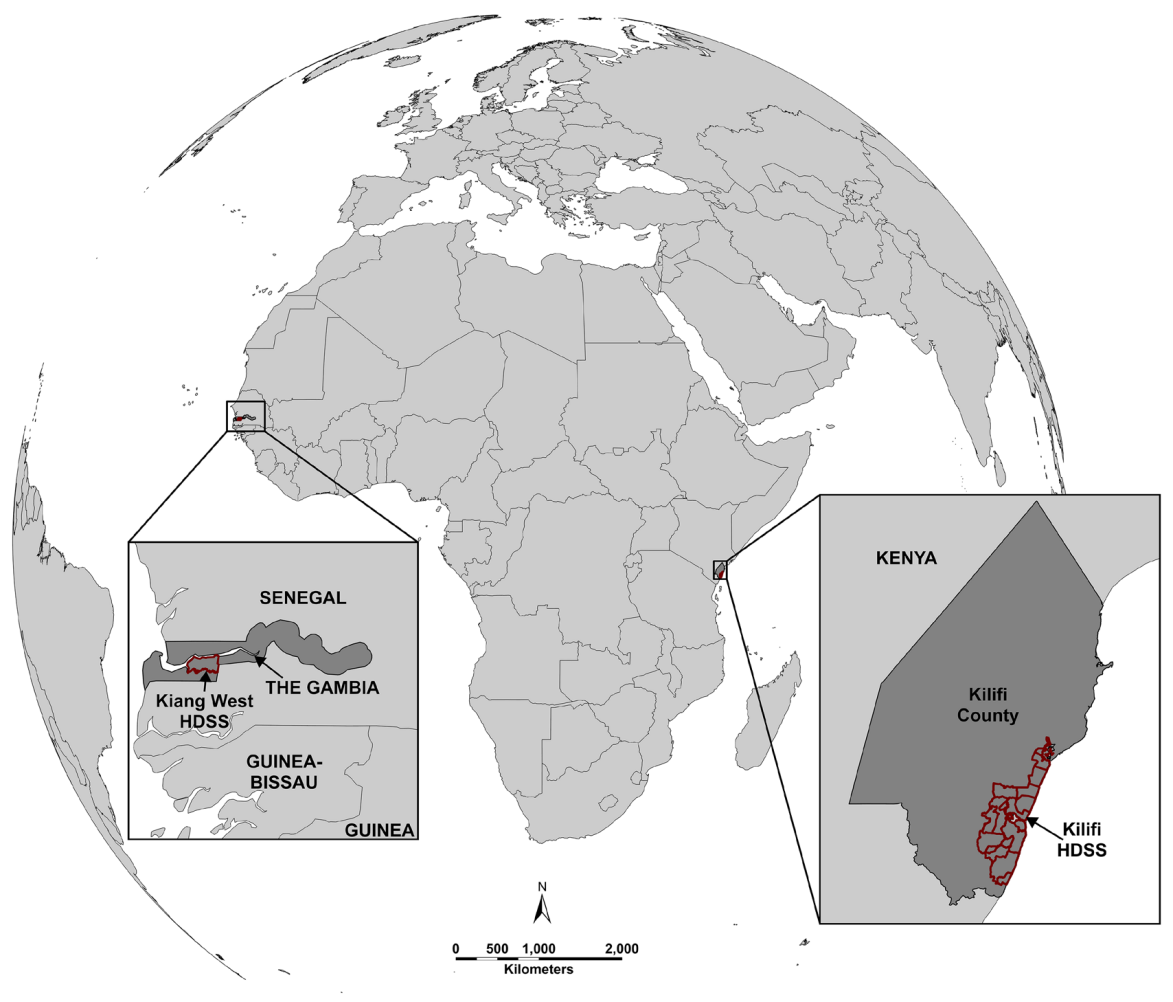
Map showing location of study sites. West Kiang Health and Demographic Surveillance System (The Gambia) and Kilifi Health and Demographic Surveillance System (Kenya). This figure is an original figure produced by the authors for this article.

Activities will take place across two rural sites, one in East Africa ( Figure 1). Both settings have a high burden of hypertension, as well as a well-developed research infrastructure to facilitate our work.

Kilifi County is one of the poorest regions in Kenya ^40^. The Kilifi Health and Demographic Surveillance System (KHDSS), covers an area of 900 km ^2^ and has a population of around 300,000 people. The prevalence of hypertension is 26% and only 3% of individuals have their blood pressure controlled ^41^. Further evidence of the consequences of poor hypertension control is the high incidence of cardiovascular disease in the area, with more than one third of patients admitted with cardiovascular disease dying during their hospital stay ^31^.

CHWs deliver health services at the community level and work within a Community Health Unit (CHU), a health service delivery structure within a defined geographical area covering approximately 5,000 people (500–1000 households). CHWs are selected from the community they serve and where possible, are required to have completed secondary education. Each CHU is required to have approximately 10 CHWs.

The Kiang West district in The Gambia covers 750km ^2^ and includes 36 villages with a population of over 14,000 individuals. It is one of the poorest regions in the country with all households in the lowest or low wealth groups. Hypertension prevalence in this region is estimated to be 40%, of whom at least 71% are undiagnosed, and only 4% controlled ^42^. The health service delivery in the region is coordinated through the Regional Health Team of the Lower River Region (Mansakonko Administrative Area). The community health nurses (CHNs) deliver services in a defined village cluster within the district. There are 4 CHNs in Kiang West with each handling 7–9 villages. CHNs work with and supervise the village health workers (VHW) who are selected by their communities.

### Study population and sample procedures

We will select an age-stratified random sample of participants aged ≥30 years from the population registers of the Kiang West Health and Demographic Surveillance System (KWHDSS, the Gambia) and Kilifi Health and Demographic Surveillance System (KHDSS, Kenya) ^43, 44^. Half of the study population will be selected from The Gambia and half from Kenya. The following age strata will be used: ≥30 to <40, ≥40 to <50, ≥50 - <60, ≥60 - <70 and ≥70y.

**Figure 2.**
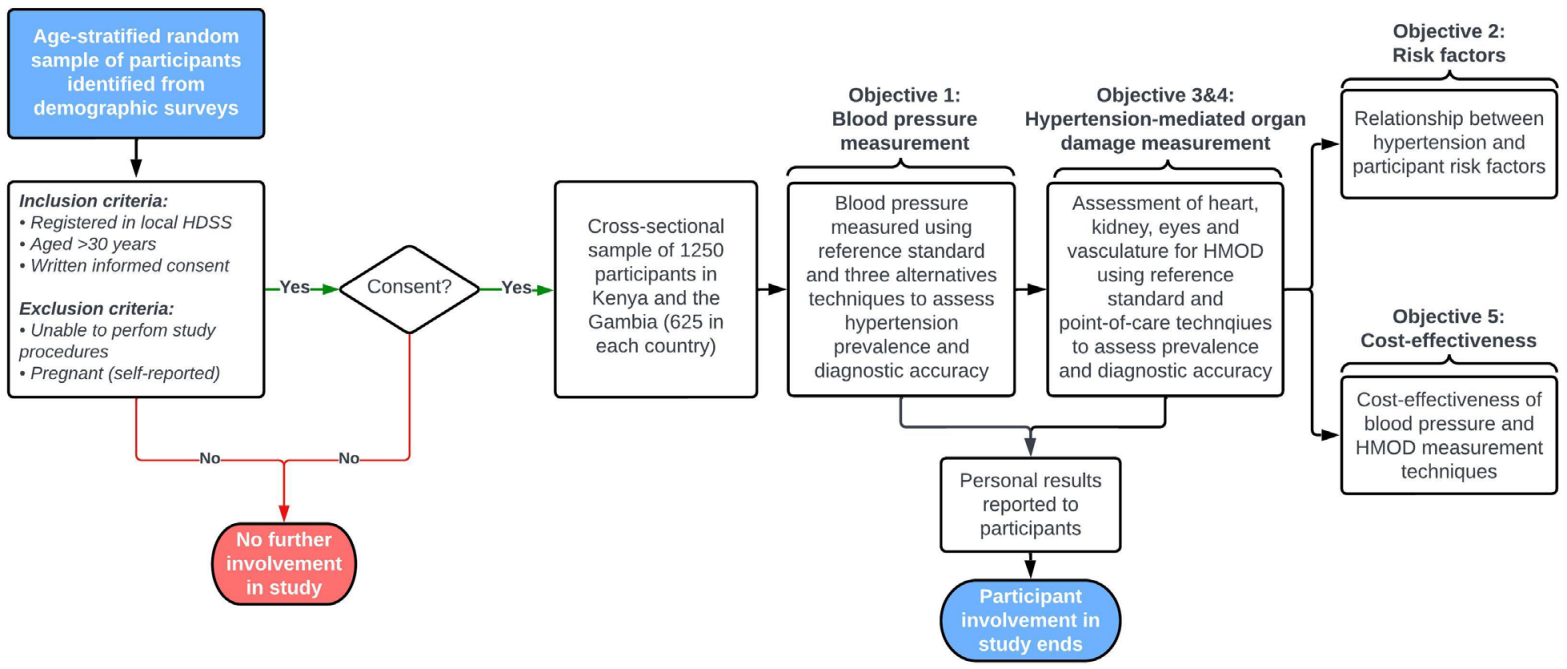
Flow diagram showing participant recruitment, study procedures and objectives.

Pregnant women will be excluded due to technical difficulties in conducting some of the study measurements such as the echocardiogram and while gestational hypertension remains important it is beyond the scope of our aims and objectives. The participant recruitment approach is shown in Figure 2.

*Inclusion criteria:*

•   Registered in population registers of the KWHDSS (The Gambia) or KHDSS (Kenya)

•   Aged ≥30 years at the time of enrolment in the study

•   Able to provide written informed consent to participate in the study

*Exclusion criteria*

•   Pregnant women (self-reported)

### Study aim and objectives

We aim to improve the evidence base for diagnosis and risk estimation for a CHW-led community-centred hypertension programme in two rural communities in SSA.

We have the following specific objectives:

1. To determine the accuracy of three alternative blood pressure measurement methods relative to the reference standard and establish the prevalence of hypertension
2. To determine the relationship between systolic blood pressure and potential risk factors
3. To determine the prevalence of HMOD
4. To determine the accuracy of innovative POC technologies to identify patients with HMOD
5. To estimate the likely cost-effectiveness of different combinations of blood pressure and HMOD measurements to initiate pharmacological treatment in people with hypertension

### Sample size

The overall sample size for the study is based on the assessment of hypertension prevalence. Assuming a prevalence of hypertension of 25–35%, as detected by the reference standard method (24-hr-ABPM), we will require 832–1003 participants to generate validity parameters for hypertension prevalence with a precision of ±3% overall and ±5% at each of the two sites ^24, 41^. We have made a provision for loss of data in 25% of participants due to poor data quality, taking the total number of participants required to 1250, equally divided between Kilifi (N=625) and Kiang West (N=625). This overall sample size will provide sufficient statistical power to address the other aims stated above.

In our assessment of BP and risk factors, and assuming for illustrative purposes that 10% of individuals will have any of the putative factors and that the standard deviation of systolic blood pressure will be 15 mmHg, then 1,003 participants will provide at least 80% statistical power for an alpha of 0.05 to detect a 4mm Hg difference in systolic BP in those with and without the exposure of interest.

Assuming a prevalence of hypertension detected by the reference standard method (24-hr-ABPM) of 30%, we estimate that approximately 300 participants from those recruited in Objective 1 will be classified as hypertensive. With 300 hypertensive patients, we would be able to estimate the confidence interval for a conservative HMOD prevalence of 35% with lower and upper intervals of 29.7% and 40.4% respectively, and that we will have over 95% power for an alpha of 0·05 to test the null hypothesis that the prevalence of HMOD is below 25%. To further explore the difference in the prevalence of HMOD in the hypertensive and general population, we intend to assess all participants recruited into the study for HMOD.

When measured with the reference standard techniques, we expect a 35% prevalence of HMOD among the 300 estimated hypertensive participants and approximately 5% prevalence in the remaining non-hypertensive participants. This will provide us with a total available sample of 153 participants with HMOD. Assuming at least 85% sensitivity of detection of HMOD by the POC strategies we will have at least 80% power for an alpha of 0.05 to test the null hypothesis that the sensitivity is below 75%. As above, we will conduct POC HMOD assessments on all participants.

### Study procedures

***Baseline assessments*.** Baseline assessments will collect information including demographics, household assets, anthropometrics, diet, physical activity, history of hypertension and medication use for hypertension and other diseases ( Table 3).

***Blood pressure assessment*.** We will conduct a cross-sectional study over two weeks to determine the accuracy of three BP measurement methods compared to the reference standard of 24-hour Ambulatory BP Measurement (24-hr-ABPM). The alternative BP measurement methods are: 1) Attended Automated BP Measurements (aABPM); 2) Unattended Automated BP Measurements (uABPM); and 3) Home-Based BP Measurement (HBPM). Details for all BP measurement methods are summarised in Table 1 and Table 3.

All BP measurements will be conducted by CHWs trained by the study team to perform the blood pressure measurements based on study SOPs. The competencies of the CHWs will be assessed by the study team prior to data collection and at regular intervals during data collection. The CHWs will then collect blood pressure measurements in a random order at participants’ homes. The random order of blood pressure measurements for each of the participants will be set using a computer-generated randomisation list produced prior to measurements starting.

**Figure 3.**
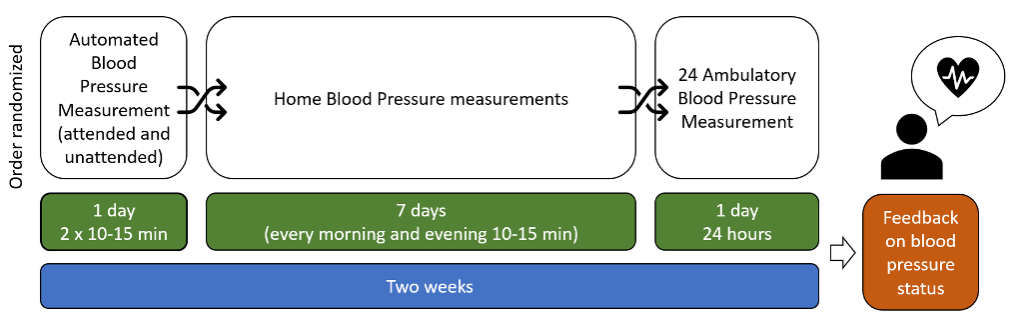
Schematic overview of blood pressure measurements performed in each participant.

Each participant is expected to have completed all four BP measurements over a two-week period. A schematic overview of the procedure is shown in Figure 3. We will follow relevant international cardiological society guidelines for conducting all BP measurements ^45– 47^.

**Figure 4.**
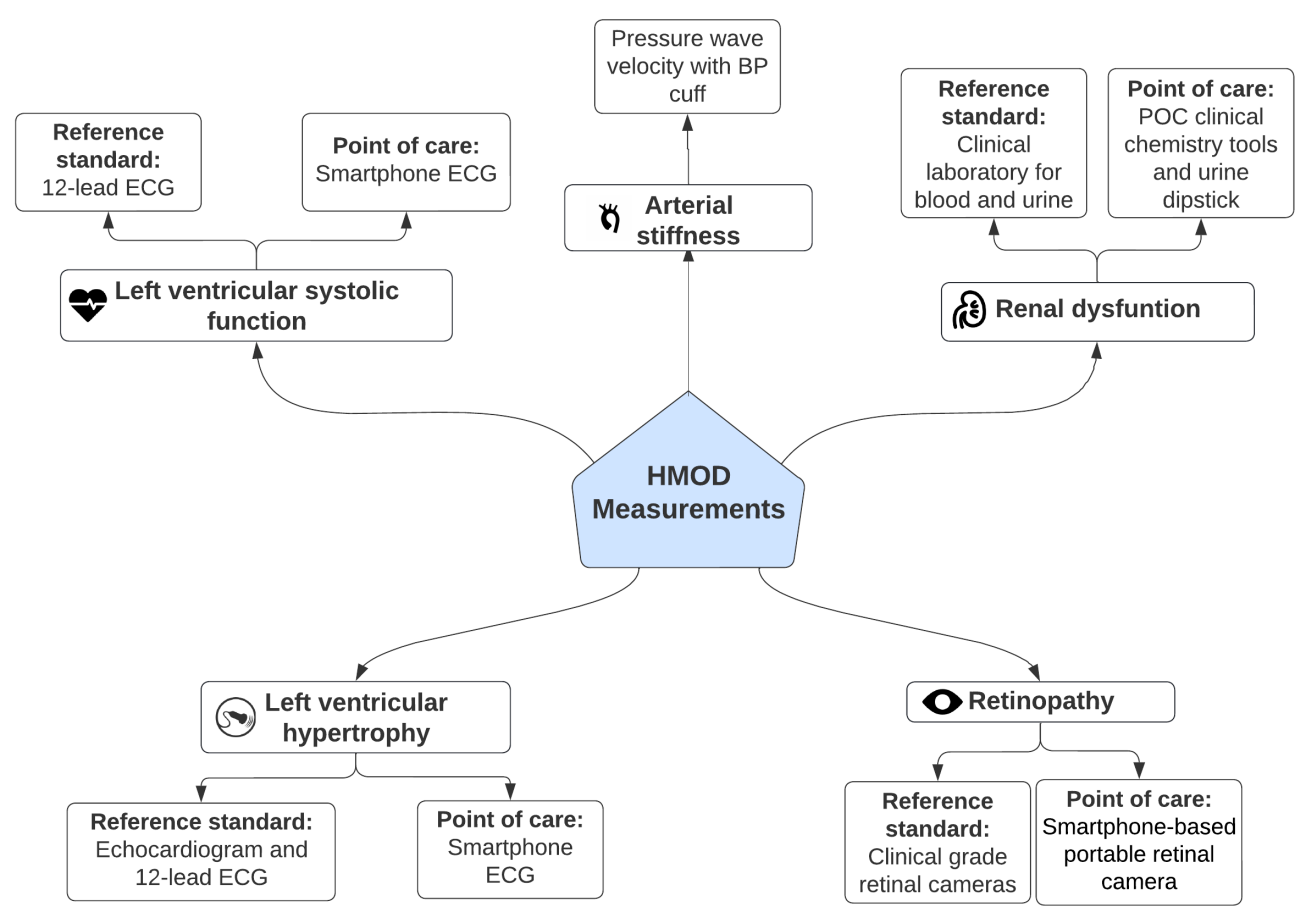
Schematic overview of hypertension-mediated organ damage measurements performed in each participant for Objectives 3 and 4.

***Hypertension mediated organ damage assessment*.** We will measure HMOD in the following organ systems: heart, eye, kidney, and vasculature. All measurements will be conducted during an in-clinic visit. The prevalence of HMOD will be assessed using reference-standard clinical assessments. In addition, we will validate the accuracy of POC assessments for HMOD compared to the reference-standard methods for the heart, eyes, and kidney. For all HMOD assessments, a detailed SOP will be used to ensure consistency between both study sites (Kenya and The Gambia) and staff will be trained in the relevant competencies. HMOD definitions in all categories will follow existing clinical guidelines ^22^. These assessments are summarised here and described in Figure 4 and Table 2 and Table 3.

**Table 2.** Overview of the methods used to measure hypertension mediated organ damage.

Type of hypertension mediated organ damage	Reference-standard assessment technique	Point-of-care assessment technique
**Left ventricular hypertrophy**	*Transthoracic echocardiogram and 12-lead ECG*	*Smartphone based 6-lead ECG*
**Left ventricular systolic function**	*Transthoracic echocardiogram*	*Smartphone based 6-lead ECG*
**Hypertensive Retinopathy**	*Clinical grade retinal cameras*	*Smartphone-based portable retinal camera*
**Renal dysfunction**	*Clinical laboratory assessment of whole blood * *and urine*	*Point-of-care clinical chemistry devices and * *urine dipstick*
**Arterial stiffness**	*Blood pressure cuff-based measurement of pulse* *wave velocity*	*Not applicable*

**Table 3.** Summary of all planned study procedures.

	Visit 1: Consent and baseline	Visits 2–4: BP measurements	Visit 5: HMOD assessment	Visit 6: Results feedback
**Eligibility assessment**	X			
**Informed consent**	X			
**Baseline questionnaires** ** - Clinical history** ** - Demographic variables** ** - Anthropometrics** ** - Treatment BP** ** - Household assets** ** - Dietary history** ** - Physical activity**	X		X	
**24-hr-ABPM**		X		
**aABPM**		X		
**uABPM**		X		
**HBPM**		X		
**BP measurement ** **acceptability questionnaire**			X	
**12-lead ECG**			X	
**Transthoracic ** **echocardiogram**			X	
**Clinical grade retinal ** **scanning**			X	
**Clinical laboratory ** **assessment of whole blood** ** and urine**			X	
**Urine dipstick**			X	
**Smartphone-based ECG**			X	
**Smartphone-based** ** fundoscopy**			X	
**Point-of-care clinical ** **chemistry**			X	
**Blood pressure cuff ** **measurement of pulse wave ** **velocity**			X	
**aABPM verification**			X	
**uABPM verification**			X	
**Participant result feedback**				X

**Reference standard HMOD assessment**

In the heart, left ventricular systolic dysfunction will be assessed with a transthoracic echocardiogram (TTE), and left ventricular hypertrophy will be assessed with a TTE and 12-lead electrocardiogram (ECG). In the eye, hypertensive retinopathy will be assessed from retinal images captured by a fixed retinal camera and graded for hypertensive retinopathy by trained staff. Kidney dysfunction will be assessed using clinical laboratory assessment of blood and urine to measure serum creatinine levels and estimate glomerular filtration rate (eGFR) and glomerular function using urine albumin:creatinine ratio and proteinuria. Finally, we will assess pulse wave velocity and arterial stiffness using a specialised blood pressure cuff (Arteriograph, TensioMed, Hungary).

**Point-of-care HMOD assessment**

Point-of-care assessment of left ventricular systolic dysfunction and left ventricular hypertrophy will be assessed using a mobile phone based 6-lead ECG (KardiaMobile, AliveCor, United States of America) coupled with artificial intelligence (AI) tools developed for ECG analysis ^48^. In the eye, we will assess hypertensive retinopathy using a handheld, mobile phone based, non-mydriatic retinal camera (Remidio, India). Retinal image grading will be conducted by the same staff who graded the reference standard images. Finally, kidney dysfunction (defined as serum creatine levels, eGFR and glomerular function) will be measured using urine dipsticks, and POC clinical chemistry devices (Abbott iStat, Abbott, United States).

***Genetic sampling*.** For the genetic analyses, DNA will be extracted from whole blood samples using Qiagen DNA Blood Mini kit. DNA extraction will make use of blood samples drawn for HMOD measurement and no new blood sample is required. We will then use PCR to determine presence of the genotypes of interest, including malaria and trypanosomiasis protective polymorphisms (Dantu, SCT, APOL1 or APOL2) ^36, 37^. These analyses will be conducted in KWTRP.

***Health economics assessment*.** We will use the human capital approach to collect primary data on patient costs including the opportunity cost of time spent administering blood pressure-monitoring methods, missing productive activity and any out-of-pocket costs incurred for undergoing study procedures. Health system cost data will be collected using a micro-costing, ingredient-based approach. Data will be collected from a structured questionnaires administered to three CHWs from each study site, structured observations of CHW and user time spent on BP and HMOD measurement, and a survey using a structured questionnaire to 200 individuals diagnosed with hypertension ^49^.

### Statistical analysis plans

A detailed statistical analysis plan will be produced before data collection is completed and uploaded to a public repository. A summary of statistical analysis approaches that will be taken for this study is outlined here.

***Hypertension prevalence*.** We will estimate sensitivity; specificity; positive and negative predictive values; and the positive and negative likelihood ratios of each BP measurement approach compared to the reference standard 24-hour BP measurement method.

***Identifying hypertension risk factors*.** The primary outcome will be hypertension, defined as a BP of >140/90mmHG using 24-hour BP measurement, or ongoing therapy with anti-hypertensives. We will determine the relationship between the candidate risk factors and blood pressure and hypertension using multivariate linear and logistic regression methods as appropriate. We will include confounders in the regression models based on results of univariate models and existing literature on the subject.

***Prevalence of organ damage*.** We will analyse the overall prevalence of HMOD and report age- and sex-standardised and stratified prevalence rates. We will also analyse prevalence rates stratified by type of HMOD (left ventricular hypertrophy, impaired left ventricular systolic function, renal dysfunction, hypertensive retinopathy, or arterial stiffness). Finally, we will construct logistic regression models to identify the participant parameters associated with presence of HMOD.

***Accuracy of POC diagnostic tools*.** The diagnostic accuracy of the POC assessments will be estimated using sensitivity; specificity; positive and negative predictive values; and positive and negative likelihood ratios of each POC parameter compared to the conventional measurement. These comparisons will be:

1) Left ventricular systolic function: Smartphone-based ECG (analysed with Mayo Clinic trained AI algorithms) compared to transthoracic echocardiogram (reference standard).
2) Left ventricular hypertrophy: Smartphone-based ECG (analysed with validated and trained AI algorithms) compared to 12-lead ECG and transthoracic echocardiogram (reference standard).
3) Hypertensive retinopathy: Smartphone-based retinal imaging compared to clinical grade retinal camera (reference standard) in detecting HMOD.
4) Renal dysfunction: POC clinical chemistry and urinalysis dipstick compared to conventional clinical laboratory assessment of blood and urine markers for renal function

***Cost effectiveness analysis*.** We will model the comparative cost-effectiveness of different decision-making approaches for initiating treatment of hypertension. We will define cost-effectiveness by comparing the costs (to be obtained from primary data collection) and outcomes (to be obtained from secondary sources) of implementing the four different BP measurement approaches, as well as the reference and PoC assessments of HMOD, to guide initiation of treatment. This analysis will be used in developing the community-centred programme at a later stage of the project.

### Ethical considerations

***Approval for research involving humans*.** Ethical approval for this research has been given by the KEMRI Scientific & Ethics Review Unit (SERU4620; 6 February 2023), the MRCG Ethics Committee and the LSHTM Ethics Committee (28276; 6 March 2023).

***Consent*.** We will obtain written informed consent from all the study participants. CHWs will be trained in the consenting procedures and will be responsible for obtaining consent. We will put in place all the necessary measures to remove potential barriers for informed consent, including language, literacy, and pressure to participate to access care.

All study participants will be provided with comprehensive information about the study in the language they are most comfortable with. This information will explain the study's aims, their specific role in it, and how data being collected will be used. Where a participant is illiterate an independent witness will be present to ensure the correct study information is presented to the participant. This witness will sign the consent form as well.

It will be made clear that participation in any part of the research will be entirely voluntary, will not influence future care and that participants will be free to withdraw at any stage, without giving a reason.

***Withdrawals*.** Participants may withdraw from the study at any time, for any reason. The study team may withdraw participants if they are no longer contactable or if they develop conditions that would exclude them from the study, including a lack of capacity to consent.

***Compensation*.** Participants will not be compensated for home blood pressure measurement visits as they will not disrupt daily activities and will take less than 15 minutes. Participants will be compensated for their time and expenses to attend hospital visits. For each visit, participants will be reimbursed 500 Kenyan shillings or 250 Gambian dalasi plus any travel expenses incurred traveling to and from the research visit.

***Urgent care referral*.** During this research, it is possible that severe medical conditions could be identified. In such cases we will arrange for urgent care and further treatment will follow the locally available standard of care.

## Discussion and expected value of results

This study is part of the wider IHCoR-Africa research group. The outcomes of this specific study will deliver the strongest evidence to date about different diagnostic approaches for hypertension in rural SSA, a unique understanding of the prevalence and characteristics of HMOD in this population, and a robust assessment of the role of innovative, low-cost, POC devices to manage hypertension in rural SSA. In addition, it will provide novel evidence of hypertension phenotypes in rural SSA. The impact of this study (together with other activities of IHCoR-Africa) will improve the long-term health outcomes of people living with hypertension in SSA.

One of the main strengths of IHCoR-Africa is the strong engagement with the community and key stakeholders. The research team has secured support from governmental organizations, civil society, scientific societies, health care providers, and patient groups. Their active engagement in IHCoR-Africa activities before and during the study will be our first step to ensuring early engagement and targeted dissemination of the results. We will present study outputs in scientific meetings of our partners including PASCAR, KCS and AESA and seminars run by the partner institutions. The IHCoR-Africa website, provides up-to-date information on the progress of the study. A formal communication and impact strategy will be developed with the support of the communication departments of participating institutions. Publications produced from IHCoR-Africa will be open-access in accordance with NIHR policy. A publication policy will be agreed by all members of the IHCoR-Africa team prior to the first paper being published.

## Study status

Ethical approvals are in place for the study. Participant consenting and data collection is underway in both sites, beginning with blood pressure measurements. Screening of HMOD with reference standard and PoC tools will commence in December 2023. Data collection is anticipated to complete by mid-2024, followed by analysis and reporting.

## Data Availability

No data are associated with this article. When the results of this study are prepared and published, anonymised data will be made available in line with the policies of the NIHR, LSHTM, KWTRP and MRCG.

## References

[1] GBD 2019 Diseases and Injuries Collaborators: Global burden of 369 diseases and injuries in 204 countries and territories, 1990–2019: a systematic analysis for the Global Burden of Disease Study 2019. *Lancet.* 2020;396(10258):1204–22. 10.1016/S0140-6736(20)30925-9 33069326 PMC7567026

[2] NCD Risk Factor Collaboration (NCD-RisC): Worldwide trends in blood pressure from 1975 to 2015: a pooled analysis of 1479 population-based measurement studies with 19·1 million participants. *Lancet.* 2017;389(10064):37–55. 10.1016/S0140-6736(16)31919-5 27863813 PMC5220163

[3] Geldsetzer P Manne-Goehler J Marcus ME : The state of hypertension care in 44 Low-Income and Middle-Income Countries: a cross-sectional study of nationally representative individual-level data from 1·1 million adults. *Lancet.* 2019;394(10199):652–62. 10.1016/S0140-6736(19)30955-9 31327566

[4] Schutte AE Botha S Fourie CMT : Recent advances in understanding hypertension development in Sub-Saharan Africa. *J Hum Hypertens.* 2017;31(8):491–500. 10.1038/jhh.2017.18 28332510

[5] Bosu WK Reilly ST Aheto JMK : Hypertension in older adults in Africa: a systematic review and meta-analysis. *PLoS One.* 2019;14(4): e0214934. 10.1371/journal.pone.0214934 30951534 PMC6450645

[6] Nulu S Aronow WS Frishman WH : Hypertension in Sub-Saharan Africa: a contextual view of patterns of disease, best management, and systems issues. *Cardiol Rev.* 2016;24(1):30–40. 10.1097/CRD.0000000000000083 26284525

[7] Ataklte F Erqou S Kaptoge S : Burden of undiagnosed hypertension in Sub-Saharan Africa: a systematic review and meta-analysis. *Hypertension.* 2015;65(2):291–8. 10.1161/HYPERTENSIONAHA.114.04394 25385758

[8] Wozniak G Khan T Gillespie C : Hypertension control cascade: a framework to improve hypertension awareness, treatment, and control. *J Clin Hypertens (Greenwich).* 2016;18(3):232–9. 10.1111/jch.12654 26337797 PMC5049660

[9] Herbst AG Olds P Nuwagaba G : Patient experiences and perspectives on hypertension at a major referral hospital in rural southwestern Uganda: a qualitative analysis. *BMJ Open.* 2021;11(1): e040650. 10.1136/bmjopen-2020-040650 33408202 PMC7789452

[10] Lasco G Mendoza J Renedo A : *Nasa dugo* (‘It’s in the blood’): lay conceptions of hypertension in the Philippines. *BMJ Glob Health.* 2020;5(7): e002295. 10.1136/bmjgh-2020-002295 32646854 PMC7351273

[11] Lynch HM Green AS Nanyonga RC : Exploring patient experiences with and attitudes towards hypertension at a private hospital in Uganda: a qualitative study. *Int J Equity Health.* 2019;18(1): 206. 10.1186/s12939-019-1109-9 31888767 PMC6937689

[12] Manavalan P Minja L Wanda L : “It’s because I think too much”: perspectives and experiences of adults with hypertension engaged in HIV care in northern Tanzania. *PLoS One.* 2020;15(12): e0243059. 10.1371/journal.pone.0243059 33270765 PMC7714125

[13] Naanyu V Vedanthan R Kamano JH : Barriers influencing linkage to hypertension care in Kenya: qualitative analysis from the LARK hypertension study. *J Gen Intern Med.* 2016;31(3):304–14. 10.1007/s11606-015-3566-1 26728782 PMC4762819

[14] Heller DJ Kumar A Kishore SP : Assessment of barriers and facilitators to the delivery of care for Noncommunicable Diseases by Nonphysician Health Workers in Low- and Middle-Income Countries: a systematic review and qualitative analysis. *JAMA Netw Open.* 2019;2(12): e1916545. 10.1001/jamanetworkopen.2019.16545 31790570 PMC6902752

[15] Cappuccio FP Miller MA : Cardiovascular Disease and hypertension in Sub-Saharan Africa: burden, risk and interventions. *Intern Emerg Med.* 2016;11(3):299–305. 10.1007/s11739-016-1423-9 27001886 PMC4820479

[16] Joshi R Alim M Kengne AP : Task shifting for Non-Communicable Disease management in Low and Middle Income Countries--a systematic review. *PLoS One.* 2014;9(8): e103754. 10.1371/journal.pone.0103754 25121789 PMC4133198

[17] Some D Edwards JK Reid T : Task shifting the management of Non-Communicable Diseases to nurses in Kibera, Kenya: *does it work*? *PLoS One.* 2016;11(1): e0145634. 10.1371/journal.pone.0145634 26812079 PMC4727908

[18] Vedanthan R Kamano JH Chrysanthopoulou SA : Group Medical Visit and Microfinance intervention for patients with diabetes or hypertension in Kenya. *J Am Coll Cardiol.* 2021;77(16):2007–18. 10.1016/j.jacc.2021.03.002 33888251 PMC8065205

[19] Schwalm JD McCready T Lopez-Jaramillo P : A community-based comprehensive intervention to reduce cardiovascular risk in hypertension (HOPE 4): a cluster-randomised controlled trial. *Lancet.* 2019;394(10205):1231–42. 10.1016/S0140-6736(19)31949-X 31488369

[20] Jafar TH Gandhi M De Silva HA : A community-based intervention for managing hypertension in rural South Asia. *N Engl J Med.* 2020;382(8):717–726. 10.1056/NEJMoa1911965 32074419

[21] Dzudie A Rayner B Ojji D : Roadmap to achieve 25% hypertension control in Africa by 2025. *Cardiovasc J Afr.* 2017;28(4):262–272. 10.5830/CVJA-2017-040 28906541 PMC5642030

[22] Williams B Mancia G Spiering W : 2018 ESC/ESH guidelines for the management of arterial hypertension: the task force for the management of arterial hypertension of the European Society of Cardiology (ESC) and the European Society of Hypertension (ESH). *Eur Heart J.* 2018;39(33):3021–3104. 10.1093/eurheartj/ehy339 30165516

[23] Whelton PK Carey RM Aronow WS : 2017 ACC/AHA/AAPA/ABC/ACPM/AGS/APhA/ASH/ASPC/NMA/PCNA guideline for the prevention, detection, evaluation, and management of high blood pressure in adults: a report of the American College of Cardiology/American Heart Association task force on clinical practice guidelines. *J Am Coll Cardiol.* 2018;71(19):e127–e248. 10.1016/j.jacc.2017.11.006 29146535

[24] Etyang AO Sigilai A Odipo E : Diagnostic accuracy of Unattended Automated Office Blood Pressure measurement in screening for hypertension in Kenya. *Hypertension.* 2019;74(6):1490–1498. 10.1161/HYPERTENSIONAHA.119.13574 31587589 PMC7069390

[25] Jones ES Damasceno A Ogola EN : PASCAR commentary on the International Society of Hypertension global guidelines 2020: relevance to Sub-Saharan Africa. *Cardiovasc J Afr.* 2020;31(6):325–329. 10.5830/CVJA-2020-055 33404583 PMC8762770

[26] Unger T Borghi C Charchar F : 2020 International Society of Hypertension global hypertension practice guidelines. *Hypertension.* 2020;75(6):1334–1357. 10.1161/HYPERTENSIONAHA.120.15026 32370572

[27] Yuyun MF Sliwa K Kengne AP : Cardiovascular Diseases in Sub-Saharan Africa compared to high-income countries: an epidemiological perspective. *Glob Heart.* 2020;15(1): 15. 10.5334/gh.403 32489788 PMC7218780

[28] Venkitachalam L Wang K Porath A : Global variation in the prevalence of elevated cholesterol in outpatients with established vascular disease or 3 cardiovascular risk factors according to national indices of economic development and health system performance. *Circulation.* 2012;125(15):1858–69. 10.1161/CIRCULATIONAHA.111.064378 22492667

[29] Reitsma MB Fullman N Ng M : Smoking prevalence and attributable disease burden in 195 countries and territories, 1990–2015: a systematic analysis from the Global Burden of Disease Study 2015. *Lancet.* 2017;389(10082):1885–1906. 10.1016/S0140-6736(17)30819-X 28390697 PMC5439023

[30] Yusuf S Rangarajan S Teo K : Cardiovascular risk and events in 17 low-, middle-, and high-income countries. *N Engl J Med.* 2014;371(9):818–27. 10.1056/NEJMoa1311890 25162888

[31] Etyang AO Munge K Bunyasi EW : Burden of disease in adults admitted to hospital in a rural region of coastal Kenya: an analysis of data from linked clinical and demographic surveillance systems. *Lancet Glob Health.* 2014;2(4):e216–e24. 10.1016/S2214-109X(14)70023-3 24782954 PMC3986034

[32] G-CHF Investigators, Joseph P Roy A : Global variations in Heart Failure etiology, management, and outcomes. *JAMA.* 2023;329(19):1650–1661. 10.1001/jama.2023.5942 37191704 PMC10189564

[33] Lee KK Bing R Kiang J : Adverse health effects associated with household air pollution: a systematic review, meta-analysis, and burden estimation study. *Lancet Glob Health.* 2020;8(11):e1427–e1434. 10.1016/S2214-109X(20)30343-0 33069303 PMC7564377

[34] Shah ASV Stelzle D Lee KK : Global burden of atherosclerotic cardiovascular disease in people living with HIV: systematic review and meta-analysis. *Circulation.* 2018;138(11):1100–1112. 10.1161/CIRCULATIONAHA.117.033369 29967196 PMC6221183

[35] Etyang AO Kapesa S Odipo E : Effect of previous exposure to malaria on Blood Pressure in Kilifi, Kenya: a mendelian randomization study. *J Am Heart Assoc.* 2019;8(6): e011771. 10.1161/JAHA.118.011771 30879408 PMC6475058

[36] Naik RP Derebail VK Grams ME : Association of Sickle Cell Trait with Chronic Kidney Disease and albuminuria in African Americans. *JAMA.* 2014;312(20):2115–25. 10.1001/jama.2014.15063 25393378 PMC4356116

[37] Genovese G Friedman DJ Ross MD : Association of trypanolytic ApoL1 variants with kidney disease in African Americans. *Science.* 2010;329(5993):841–5. 10.1126/science.1193032 20647424 PMC2980843

[38] Ojji DB Libhaber E Atherton JJ : Risk-factor profile and comorbidities in 2398 patients with newly diagnosed Hypertension from the Abuja heart study. *Medicine (Baltimore).* 2015;94(39): e1660. 10.1097/MD.0000000000001660 26426662 PMC4616876

[39] Damasceno A Mayosi BM Sani M : The causes, treatment, and outcome of Acute Heart Failure in 1006 Africans from 9 countries: results of The Sub-Saharan Africa Survey of Heart Failure. *Arch Intern Med.* 2012;172(18):1386–94. 10.1001/archinternmed.2012.3310 22945249

[40] Adetifa IMO Bwanaali T Wafula J : Cohort profile: the Kilifi Vaccine Monitoring Study. *Int J Epidemiol.* 2017;46(3):792–792h. 10.1093/ije/dyw202 27789669 PMC5654374

[41] Etyang AO Warne B Kapesa S : Clinical and epidemiological implications of 24‐hour Ambulatory Blood Pressure Monitoring for the diagnosis of hypertension in Kenyan adults. A population‐based study. *J Am Heart Assoc.* 2016;5(12): e004797. 10.1161/JAHA.116.004797 27979807 PMC5210452

[42] Cham B Scholes S Ng Fat L : Burden of hypertension in the Gambia: evidence from a national World Health Organization (WHO) STEP survey. *Int J Epidemiol.* 2018;47(3):860–871. 10.1093/ije/dyx279 29394353

[43] Hennig BJ Unger SA Dondeh BL : Cohort profile: the Kiang West Longitudinal Population Study (KWLPS)—a platform for integrated research and health care provision in rural Gambia. *Int J Epidemiol.* 2017;46(2): e13. 10.1093/ije/dyv206 26559544 PMC5837564

[44] Scott JAG Bauni E Moisi JC : Profile: the Kilifi Health and Demographic Surveillance System (KHDSS). *Int J Epidemiol.* 2012;41(3):650–7. 10.1093/ije/dys062 22544844 PMC3396317

[45] O’Brien E Parati G Stergiou G : European Society of Hypertension position paper on Ambulatory Blood Pressure Monitoring. *J Hypertens.* 2013;31(9):1731–68. 10.1097/HJH.0b013e328363e964 24029863

[46] Mancia G Fagard R Narkiewicz K : 2013 ESH/ESC Guidelines for the management of arterial hypertension. *Arterial Hypertension.* 2013;17(2):69–168.

[47] Excellence NIfHaC: Hypertension in adults: diagnosis and management. 2011.

[48] Attia ZI Kapa S Lopez-Jimenez F : Screening for cardiac contractile dysfunction using an Artificial Intelligence–enabled Electrocardiogram. *Nat Med.* 2019;25(1):70–74. 10.1038/s41591-018-0240-2 30617318

[49] Oyando R Njoroge M Nguhiu P : Patient costs of hypertension care in public health care facilities in Kenya. *Int J Health Plann Manage.* 2019;34(2):e1166–e1178. 10.1002/hpm.2752 30762904 PMC6618067

