# Supplementary material for: Determining the optimal diagnostic and risk stratification approaches for people with hypertension in two rural populations in Kenya and The Gambia: a study protocol for IHCoR-Africa Work Package 2
Source: NIHR Open Res. 2024 May 17;3:68. Originally published 2023 Dec 21. [Version 2] doi: 10.3310/nihropenres.13509.2 (PMC11319908; doi:10.3310/nihropenres.13509.2)
Supplement: Peer review history

# Reviewer report: Reviewer response for version 2

**DOI:** 10.3310/nihropenres.14785.r31840

**Published:** 2024-05-29

**Authors:** Liangdi Xie

**Affiliations:** 1 The First Affiliated Hospital of Fujian Medical University, Fuzhou, China

The authors did not respond to the reviewer's comments and made revisions. Why did you choose the patients those over 30 years old instead of those over 18 years old as subjects? As mentioned, hypertension occurs at younger ages in SSA.

Is the study design appropriate for the research question?

Partly

Is the rationale for, and objectives of, the study clearly described?

Yes

Are sufficient details of the methods provided to allow replication by others?

Partly

Are the datasets clearly presented in a useable and accessible format?

Yes

Reviewer Expertise:

Hypertension; Pulmonary hypertension

I confirm that I have read this submission and believe that I have an appropriate level of expertise to confirm that it is of an acceptable scientific standard.

# Reviewer report: Reviewer response for version 1

**DOI:** 10.3310/nihropenres.14659.r31115

**Published:** 2024-03-07

**Authors:** Destaw Fetene Teshome

**Affiliations:** 1 University of Gondar, Gondar, Ethiopia

**Determining the optimal diagnostic and risk stratification** **approaches for people with hypertension in two rural** **populations in Kenya and The Gambia: a study protocol for** **IHCoR-Africa Work Package 2 [version 1; peer review: 1** **approved with reservations]**

**Reviewer responses**

 The authors identified one of the most significant global public health issues, hypertension. Determining the best diagnostic and risk stratification approaches for people with hypertension is critical because early detection allows for timely intervention, including lifestyle changes and appropriate treatment, to prevent hypertension progression and associated complications. I found the protocol is well written and contributes to the scientific community and the community at large.  However, I have some concerns that will be addressed in the revised manuscript.

**Abstract**

- line 5. Is task shifting or task sharing better suited for community health workers? Is it possible to shift all diagnostic activities from trained health professionals to community health workers in an African setting such as Kenya?

**Introduction **

- Applicability of the “Home BP Measurement” in rural settings where majority of the population cannot read and write is questionable.

**Methodology**

 Patient and Public Involvement

- I do not think the sentence ”Throughout the course of ***the we*** will meet with CHWs and patient groups, to under- stand their experiences as the project is implemented” is completed. This should be revised and addressed in the revised version.

Inclusion criteria

- Aged ≥30 years at the time of enrolment in the study could not be the inclusion criteria as you are described in the study population sub section. I recommend you delete it in the revised version of the protocol.

Is the study design appropriate for the research question?

Yes

Is the rationale for, and objectives of, the study clearly described?

Yes

Are sufficient details of the methods provided to allow replication by others?

Yes

Are the datasets clearly presented in a useable and accessible format?

Yes

Reviewer Expertise:

Noncommunicable disease research

I confirm that I have read this submission and believe that I have an appropriate level of expertise to confirm that it is of an acceptable scientific standard.

# Response

**Published:** 2024-05-07

**Authors:** Alexander Perkins

**Affiliations:** NCDE, London School of Hygiene & Tropical Medicine, London, UK

Many thanks for the thoughtful review. We have provided our response below, and have updated the manuscript with minor changes as needed. 

1. This is an excellent point and we have rephrased the abstract to refer to 'task sharing' to represent more accurately what happens in practice, as the reviewer suggests.
2. We agree that there will be challenges to implementing Home BP Measurement in this context. However, understanding the challenges to implementation is a key outcome from this study and we believe that including this commonly used BP measurement technique is important.
3. Our thanks for pointing out this error in the text. The sentence has a missing word and we have revised to “Throughout the course of the study we will meet with CHWs and patient groups, to understand their experiences as the project is implemented.”
4. We acknowledge that there is some redundancy here. However, we maintain that it is important to clearly state the age limit in the inclusion and exclusion criteria to clearly define the characteristics of the population. 

# Reviewer report: Reviewer response for version 1

**DOI:** 10.3310/nihropenres.14659.r31118

**Published:** 2024-02-21

**Authors:** Liangdi Xie

**Affiliations:** 1 The First Affiliated Hospital of Fujian Medical University, Fuzhou, China

This study elaborated the detail procedure of the community-centred programme to improve the evidence base for diagnosis and risk estimation for a community-centred hypertension programme in two rural settings in Sub-Saharan Africa (SSA). This study is meaningful in improving the diagnosis accuracy and treatment of hypertension in the SSA. There are some questions that have not been clarified very clearly.

 1. No indication of whether the enrolled population had received antihypertensive treatment. According to your inclusion criteria, there may be some hypertension patients who had already received medicine therapy. To ensure accurate blood pressure observations, it is advisable that anti-hypertensive medications cannot be adjusted during the follow-up period, thereby eliminating any potential impact of medication adjustments.

1. Left ventricular systolic dysfunction was assessed with a transthoracic echocardiogram (TTE), and left ventricular hypertrophy was assessed with a TTE and 12-lead electrocardiogram (ECG). What was the definition of left ventricular hypertrophy and left ventricular systolic dysfunction, respectively?
2. It is recommended to use the echocardiography to evaluate these parameters?
3. What did you do when blood pressure significantly elevated during the follow-up period in patients with hypertension who require immediate medications?
4. Please introduce the point-of-care more specifically? And please briefly elaborate on the feasibility of the popularization of this smart device in the SSA.
5. Why did you choose the patients those over 30 years old instead of those over 18 years old as subjects? As mentioned earlier, the patients here were younger.
6. As mentioned in page 6, hypertension prevalence was estimated to be 40% in this region. Why did the authors assume a prevalence of 25-35% in sample calculation? It seems that number of lost visits is not considered in sample calculation.
7. Please describe the specific procedures of measuring PWV by BP cuff in the methods.
8. Pregnant women were excluded not due to technical difficulties in conducting some of the study measurements such as the echocardiogram, but because of the particularity of their hemodynamics, which was not the key point of this study.

Is the study design appropriate for the research question?

Partly

Is the rationale for, and objectives of, the study clearly described?

Yes

Are sufficient details of the methods provided to allow replication by others?

Partly

Are the datasets clearly presented in a useable and accessible format?

Yes

Reviewer Expertise:

Hypertension; Pulmonary hypertension

I confirm that I have read this submission and believe that I have an appropriate level of expertise to confirm that it is of an acceptable scientific standard, however I have significant reservations, as outlined above.

# Response

**Published:** 2024-05-07

**Authors:** Alexander Perkins

**Affiliations:** NCDE, London School of Hygiene & Tropical Medicine, London, UK

We would like to express our thanks to the reviewer for their time and their comments on this article. Below we have provided our responses. Where needed, small revisions to the text of the article have been made. 

1. This paper summarises the design of the IHCoR-Africa study. As a result, we do not yet have data on the use of anti-hypertensive medication in the enrolled population. We agree that this is a key piece of information, and are actively collecting it in our database. We will report the use of anti-hypertensive medication as part of the primary results paper once the study has concluded. To account for use of anti-hypertensives, will examine accuracy of BP measurements for those on medication and those not.
2. As mentioned in the section "Hypertension mediated organ damage assessment", we will follow international guidelines on HMOD definitions (including for LVSD and LVH) including those published by the European Society of Cardiology, the International Society for Hypertension and the American Heart Association. The specifics of the definitions used will be included when we report the results of this work.
3. Yes. Treatment guidelines, such as the ESC Hypertension guidelines, recommend transthoracic echocardiogram as a diagnostic approach for left ventricular systolic dysfunction and left ventricular hypertrophy. 
4. In the section titled "Urgent care referral" we detail arrangements for referral to urgent care for participants who require it during the study. Specific information of what happened and the numbers who required this will be reported at the end of the study once we have results. 
5. This study includes several point of care devices and they are detailed in Figure 4 and Table 2. As the reviewer highlights, the feasibility of using these devices in this setting is a key outcome and will be an important result from this study.
6. We chose subjects over 30 in order to increase the efficiency (and lower the cost) of the study as prevalence rates of hypertension are higher in those over 30 years. There is active work in sub-Saharan Africa is exploring hypertension in younger people (such as the CHIEDZA study: https://doi.org/10.1016/S2352-4642(23)00287-0).
7. Estimates of hypertension vary by the study site. In Kilifi the estimate is 26% and in the Gambia it is 40%. As a result we used a conservative estimate of 25-35%. We do consider loss to follow-up in our sample size calculations.
8. Pulse wave velocity will be measured using the Arteriograph device (Tensiomed Inc, Budapest, Hungary). We have amended the text “Reference Standard HMOD assessment” to more clearly indicate this. Details of the specific procedure will be included in the results paper once this study is complete.
9. Pregnant women were excluded because of technical difficulties. For example for echocardiography, certain views are difficult to acquire due to pregnancy. In addition given the significant haemodynamic and vascular changes that occur during pregnancy, gestational hypertension was out of scope for our research programme.
